# Flu shot in the era of COVID-19 vaccination: findings from a research hospital of Rome: Domenico Pascucci

**DOI:** 10.1093/eurpub/ckac131.391

**Published:** 2022-10-25

**Authors:** D Pascucci, MC Nurchis, M Sapienza, A Lontano, E Marziali, F Castrini, W Ricciardi, G Damiani, P Laurenti

**Affiliations:** 1 Università Cattolica del Sacro Cuore, Rome, Italy; 2 Fondazione Policlinico A. Gemelli IRCCS, Rome, Italy

## Abstract

Influenza represents a major burden for public health. Healthcare workers (HCWs) are a priority target group for flu vaccination. During the COVID-19 pandemic, when SARS-CoV-2 vaccines were not yet available, susceptibility to influenza vaccination especially by HCWs increased. The aim of this study is to analyze the flu vaccination coverage among HCWs and to study which factors affected their adherence given the concomitant COVID-19 vaccination. The retrospective study was conducted in an Italian research hospital from October 2021 to January 2022. A total of 7,048 individuals was included. Age class, gender and job category variables were analyzed. Statistically significant differences among groups were tested through χ2 test. Univariate and multivariate analyses (p < 0,005) were performed to assess differences towards vaccination attitude. The flu vaccination coverage rate was 24.6%. Among the selected job categories, 29.8% of physicians, 19.9% of nurses and 19.7% of other HCWs were vaccinated with a statistically significant decrease (p < 0.001) across all categories respect with the last campaign. The findings of the logistic regression depicted that the 40-59 years old age class, compared with the youngest age class (OR 1.30, 95% CI 1.12-1.43) as well as being physician (OR 2.79, 95% CI 1.87-3.41) with the respect to being nurses, had a higher adherence to vaccination. Interestingly, being male, is associated with a statistically significant reduction (OR 0.71, 95% CI 0.59-0.87) in vaccination uptake. Study findings showed a several decline in the flu vaccination coverage comparing with previous campaigns, probably due to the concomitant administration of the booster dose against SARS-CoV-2. This alarm should not be underestimated and requires timely and innovative organizational approaches (i.e., combined vaccine). Further studies are needed to analyze the reasons for this poor adhesion and the strategies to be adopted to increase the awareness of the HCWs.

**Key messages:**

• Reaching high coverage rates and restore a positive trend for the future campaign for flu vaccination it is essential strategy to protect HCWs themselves, their patients and the hospital community.

• Decision-makers should implement consistent communication strategies to lessen vaccine hesitancy among HCWs.

